# Effects of Debriefing on Motivation and Reflective Thinking of Nursing Students during In-School Practicum Using a Flipped Learning Model

**DOI:** 10.3390/healthcare10122552

**Published:** 2022-12-16

**Authors:** Sisook Kim, Yedong Son

**Affiliations:** 1 Department of Nursing, Hwasung Medi-Science University, Hwaseong-si 18274, Republic of Korea; 2 College of Nursing, Woosuk University, Wanju-gun 55338, Republic of Korea

**Keywords:** debriefing, flipped learning, nursing education, nursing students, reflective thinking

## Abstract

Debriefing and flipped learning have been determined to be useful strategies for nursing education. Recently, it has been reported that applying debriefing and flipped learning together was helpful for educational outcomes. The objective of this study was to compare learning motivation and reflective thinking before and after debriefing during nursing practicums that applied flipped learning. We implemented a quasi-experimental procedure in the form of a pretest-posttest non-equivalent control group design (1 September to 22 October 2021). The participants comprised 63 nursing students in South Korea (33 in the experimental group and 30 in the control group). Each group took part in a nursing practice class, wherein the experimental group engaged in debriefing using flipped learning, while the control group engaged only in flipped learning. We then examined academic motivation and reflective thinking in both the groups, and found that reflective thinking was significantly higher in the experimental group (53.67 ± 5.71) versus the control group (50.80 ± 4.69) (t = 2.165, *p* = 0.034). However, there were no differences in learning motivation between the two groups (t = 1.864, *p* = 0.067). In sum, this study confirmed the benefits of this new teaching approach, in which debriefing and flipped learning were integrated and incorporated into a nursing practice class. Given our promising results, this approach can be applied in various cases to strengthen the clinical practice skills of nursing students.

## 1. Introduction

### 1.1. Study Rationale

Unlike traditional learning, the flipped learning approach conveys information outside the classroom, where students complete lessons through active participation and interactions with their instructors and peers [1]. Following the introduction of relevant information and communication technologies, students are given a preview lesson using e-learning materials and lecture videos provided by their instructor, and thereafter, a face-to-face lesson that implements practical problem-solving and in-depth learning [2]. Flipped learning has been applied in many fields, including natural science, engineering and technology, social science, and public health. Given its medium effect size on both academic achievement and motivation, regardless of the specific study area or instructor, flipped learning is actively used at various colleges [3]. It is especially useful under the constraints of the COVID-19 pandemic, as college students can flexibly take lessons that combine flipped learning and face-to-face classes, without missing lectures [4].

In nursing education, various theories need to be applied to clinical cases. Under this framework, flipped learning is considered an alternative educational approach that facilitates the application of knowledge, analysis, and assessment in lectures. Flipped learning effectively supports learning and improves the quality of education in both nursing theory and practicum classes [5]. A meta-analysis on the effects of flipped learning in public health education (including nursing) reported that the approach could strengthen due to both easy accessibility and the frequent repetition of preview lessons [6].

In flipped learning, learning motivation is an important factor influencing the effectiveness of learning [7]. For instructors to effectively perform flipped learning, active learning should be conducted to allow students to autonomously participate in pre-learning activities and reflect on those activities [8]. Higher motivation can help students immerse themselves in learning and learn new skills [9]. Therefore, it is important to maintain motivation for learning.

Reflective thinking reinterprets the meaning of experience and applies it to activities again; in nursing, it means that nurses evaluate their own nursing practice and improve their clinical practice competency for the future [10]. In an experimental study that promoted reflective thinking by conducting a learning evaluation after participating in a flipped learning class for college students who were taking a digital learning course, reflective thinking had a positive effect on students’ learning motivation and outcomes. [11]. By promoting reflective thinking, students actively participate in activities before and during classes in a flipped learning class and are able to recognize and control their own learning [11]. Therefore, in nursing education, it is necessary to examine whether learning activities that promote reflective thinking can be helpful for flipped learning classes.

Debriefing is a reflective learning process that is designed to improve and clarify thinking [12]. In nursing education, debriefing induces maximum training benefits by increasing motivation [13] and promoting the reflective thinking process [14]. It is primarily a means of integrating cyclical and reflective learning to improve learning outcomes in various military, aviation, and medical fields [15]. Although continuous efforts are made to improve the strategies used for debriefing, which are now emerging in medical simulation lessons through constant advancements [16], the process is not commonly applied outside simulation lessons in the formal capacity.

A previous study suggested that debriefing and flipped learning could be integrated to further acquire skills and knowledge [17]. Especially during the COVID-19 pandemic, nursing education had to emphasize professional career independence. As flipped learning underscores the importance of self-directed learning, it allows students to effectively acquire nursing techniques by providing sufficient time for training and feedback assessment. Further, debriefing can encourage students to reflect on their own learning experiences, and thereby adapt to rapid changes in the medical environment. To reinforce learning motivation and promote reflective thinking, this study applied debriefing to nursing practice education in conjunction with flipped learning, with the ultimate aim of both expanding this innovative educational approach and enhancing learning outcomes for nursing students.

### 1.2. Problem Statement

Flipped learning is an educational method that promotes learning motivation by allowing students to learn on their own before class and is useful for nursing theory and practicum classes [5]. By improving their reflective thinking, students become more actively involved in the flipped learning process [11]. Reflective thinking, especially, helps improve clinical practice competencies in nursing education [10] and is facilitated through debriefing [14]. Debriefing is a learning method that integrates learning and improves performance in a circular and reflective way, usually in simulation education [15]. It is rare to apply debriefing in nursing practicum classes in schools. Recently, a study reported that applying both flipped learning and debriefing methods to education can be helpful for education [17]. Therefore, it is necessary to examine whether debriefing is effective for improving learning motivation and reflective thinking by applying debriefing in nursing practicum classes in schools that use flipped learning.

### 1.3. Objective

The objective of this study was to compare learning motivation and reflective thinking before and after a debriefing in nursing practicum classes that used flipped learning. The specific objectives are as follows.

- Compare learning motivation before and after a debriefing in the experimental and control groups.
- Compare reflective thinking before and after a debriefing in the experimental and control groups.

### 1.4. Hypotheses

**Hypothesis** **1 (H1).** There is a statistically significant difference in learning motivation between the experimental group, which received debriefing education, and the control group.

**Hypothesis** **2 (H2).** There is a statistically significant difference in reflective thinking between the experimental group, which received debriefing education, and the control group.

## 2. Methods

### 2.1. Study Design

In this quasi-experimental study, we implemented a pretest-posttest non-equivalent control group design to examine how debriefing impacted academic motivation and reflective thinking, when included with flipped learning at a nursing college.

### 2.2. Participants

The participants were nursing students attending a college in Chungcheongnam-do, South Korea. The number of participants was calculated using G*Power 3.1.9.7 [18]. With a significance level of 0.05, a power of 0.8, and a median effect size of 0.5, referring to a previous study [3], 34 was the number calculated for each group, and 41 were recruited to each group to accommodate a 20% dropout rate. Excluding the missing values on surveys, the final participants in this study were 36 in the experimental group and 30 in the control group.

### 2.3. Experimental Treatment

The experimental treatment was the addition of debriefing during a six-week flipped learning class. However, the control and experimental groups both engaged in a flipped learning class consisting of: 10 min of quizzes, 30 min of lecturing and demonstration, 50 min of group training, and 10 min of review. Additionally, during the 50 min of group training, the participants in the experimental group experienced debriefing with assigned teammates (Table 1). All experimental and control groups participated in a practicum to develop basic nursing skill competencies, which was a targeted curriculum prior to clinical practice. This class practiced different nursing skills each week for 100 min in a laboratory room. These nursing skills are for the practice of new nurses, such as vital signs, nasal cannula, pulse oximeter, pre- and post-operation care, nasogastric tube feeding, and endotracheal suction.

Specifically, the flipped learning learners—the participants in the experimental and the control group—were provided with images and terms for different nursing skills every week to be learned before classes. For video learning, students learned through videos related to nursing skills provided on the Elsevier website (https://www.els-nursingskills.kr (accessed on 2 March 2021)) and were provided with related terms by instructors. Quizzes were conducted on nursing skill videos and related terms for 10 min, followed by lectures and demonstrations by the instructor for 30 min. After that, for 50 min, the experimental group had group practice and debriefing. The debriefing applied to the experimental group was as follows: For 50 min, the members took pictures of each other using mobile cameras during group practice. While replying to the videos right away, interaction and discussion were conducted within the group. The instructors took turns participating in each group and facilitated interaction and discussion about the filmed videos. Each week, the content of one group’s debriefing was discussed with feedback from all other groups. The control group practiced in groups for 50 min. Finally, for 10 min, the instructors summarized the contents of the class. The instructors took turns participating in each group to facilitate the practicum.

In-school training, or the basic nursing training curriculum, consists of a total of 12 weekly lessons, excluding one orientation and two tests. The instructors were the author and a part-time lecturer, who shared the implementation of the study and standardized the protocol for flipped learning and debriefing. Each of them taught one experimental group and one control group. For classes held during weeks 1–6, the control group solely engaged in flipped learning, while the experimental group engaged in flipped learning with debriefing. For classes held during weeks 7–12 (after completing the experiment), debriefing was added to flipped learning for the control group, thus providing opportunities for all participants to experience debriefing.

To ensure ethical research, the lesson plan was provided to all students taking the class. The research plan was introduced during week one of the orientation to encourage voluntary participation. In all cases, we obtained consent to collect and use video information that the participants recorded during this study. They also received a lecture on safety management and the protection of rights for private video information.

### 2.4. Measures and Instruments

#### 2.4.1. Demographic Characteristics

We considered a variety of demographic characteristics, including gender, age, satisfaction with one’s major, grades from the previous semester, online learning experience, flipped learning experience, and preferred digital device.

#### 2.4.2. Academic Motivation

Motivation refers to the process of initiating, guiding, and maintaining goal-orientated behavior. In learning, motivation constitutes an internal or external power that produces voluntary learning behavior. It is a critical factor for effective learning and achievement, which impacts the preference for learning and cognition [19]. In this study, we measured academic motivation via the Course Interest Survey (CIS) [20,21], which was developed using the attention, relevance, confidence, and satisfaction (ARCS) model. This tool had a total of 34 items on a 5-point Likert scale at the time of development [20,21]. In this study, a Korean version of the tool with 20 items that were validated for Korean college students was used [22]. This tool was calculated by the mean value of all items; the higher the score, the higher the learning motivation. In this study, permission was obtained from the author of this tool that was developed for Korean college students. The Cronbach’s α of each domain in the Korean version of the tool ranged from 0.5 to 0.84. [22]. In this study, the instrument received Cronbach’s α values of 0.772 and 0.803 before and after the experiment, respectively.

#### 2.4.3. Reflective Thinking

Reflective thinking—an advanced and individualized response to circumstances that present a gap between theory and practice—also refers to the cognitive activity of intentionally and objectively participating in disclosure with the self to both understand a current situation or phenomenon and act accordingly [23]. In nursing education, reflective thinking is strongly connected with the outcome of evaluating one’s self and improving clinical practice skills [10]. In this study, we measured reflective thinking via the Self-Reflection and Insight Scale (SRIS) [24], which was translated into Korean for use among nursing students in South Korea [25], thus providing a means of monitoring and evaluating individual performances to achieve specific clinical goals. The SRIS consists of 16 items, including 11 on self-reflection and five on insight. This tool was on a 5-point Likert scale, and the score ranged from 20 to 100; the higher the score, the higher the level of reflective thinking. In this study, permission was obtained from the original author and the author who translated the tool into Korean to use the Korean version of the tool. At the time of development, the scale received Cronbach’s α values of 0.91 for self-reflection and 0.87 for insight [24]. Another study reported an overall Cronbach’s α value of 0.80 for the Korean version of the SRIS [25]. In this study, the scale received Cronbach’s α values of 0.602 and 0.683 before and after the experiment, respectively.

### 2.5. Data Collection

Data were collected between 1 September and 22 October 2021. The research assistant posted a recruitment notice in the classroom, and voluntary participants filled out a structured self-report questionnaire during the pre-and post-surveys. The pretest data were collected prior to the first day of the practicum course, and posttest data were collected after completing the six-week practicum course for both the experimental and control groups. The survey data were collected anonymously, and only nicknames were indicated on the questionnaire to compare pre-and post-surveys.

### 2.6. Data Analysis

The collected data were analyzed using IBM SPSS 21.0. Demographic characteristics were described as frequencies and percentages, while academic motivation, immersion, and reflective thinking were described as means and standard deviations. We tested the reliability of both academic motivation and reflective thinking via Cronbach’s α. The homogeneity of the experimental and control groups was tested using a *t*-test. Finally, we verified differences in academic motivation, immersion, and reflective thinking before and after the experiment via the paired *t*-test.

### 2.7. Ethical Considerations

This study was approved by the IRB of the affiliated research institute (IRB No. 1041479-HR-202101-005). The researchers explained the purpose of the study and the participation method to the participants, and after confirming the intention to participate with the research assistant, written consent was obtained. Since a research assistant conducted the survey and gathered written consent, neither the researchers nor the lecturers who conducted the class could know which students participated. We explained that nicknames were used for comparison before and after the class, the anonymity of the research participants was guaranteed, and the survey content would not be used for any purpose other than research. Further, students were told that they could refuse or withdraw from participation at any time.

## 3. Results

### 3.1. Homogeneity Test on the Demographic Characteristics

The participants were sophomores at a nursing college, including 33 in the experimental group and 30 in the control group. There were no differences between the groups in gender (χ^2^ =0.814, *p* = 0.367), age (t = 0.33, *p* = 0.367), satisfaction with major (χ^2^ = 1.405, *p* = 0.495), grades from the previous semester (χ^2^ = 1.541, *p* = 0.463), or flipped learning experience (χ^2^ = 4.100, *p* = 0.129). Thus, homogeneity between the groups was verified (Table 2).

### 3.2. Homogeneity Test on the Dependent Variables between Groups

The homogeneity test on pretest scores for academic motivation and reflective thinking showed no statistically significant differences in either academic motivation (t = 1.917, *p* = 0.060) or reflective thinking (t = 1.250, *p* = 0.216) between the experimental and control groups, thus verifying homogeneity between the groups (Table 3).

### 3.3. Hypothesis Testing

First, the experimental group had higher academic motivation than the control group (3.71 ± 0.29 vs. 3.58 ± 0.26), but this difference was not statistically significant (t = 1.864, *p* = 0.067). Thus, H1 was rejected. Second, the experimental group had higher reflective thinking than the control group (53.67 ± 5.71 vs. 50.80 ± 4.69). This difference was statistically significant (t = 2.165, *p* = 0.034), which supported H2. Table 4 lists the results.

## 4. Discussion

Debriefing [13,14] and flipped learning [6] are effective educational approaches with positive effects on nursing education. However, their combined educational effects require further validation [17]. To address this gap in the literature, the current study added debriefing to in-school practicum via flipped learning, then examined how this impacted academic motivation and reflective thinking in a sample of college students.

First, there were no significant differences in academic motivation between the experimental group (3.71 ± 0.29) and the control group (3.58 ± 0.26). A study on students at police schools found that they were more motivated to train skills related to scenarios when instructors applied a debriefing using videos [26], which was inconsistent with this study. This study also applied a debriefing using peer-led, not instructor-led, filming of video debriefing, which was different from a study by Sjöberg et al. that applied a debriefing by instructors [26]. In nursing education, debriefing among peers is a method to elicit positive experiences through student-led communication of their experiences, without an instructor [27]. Successful peer debriefing can be facilitated if guidance on the principles of debriefing proceeds [28]. Therefore, it would be helpful to educate students in detail on the purpose of debriefing, debriefing evaluation tools, and feedback methods before debriefing.

A more positive result, reflective thinking was significantly higher in the experimental group (53.67 ± 5.71) versus the control group (50.80 ± 4.69). Reflection is a crucial factor in debriefing; at the same time, student-oriented debriefing activities (especially when students mutually share their opinions) can strengthen the reflection process [29]. In this study, the participants were asked to record self-videos and conduct debriefing with teammates. A previous study that analyzed the learning effects of debriefing with self-videos among nursing students found that the learning process enabled repeated learning, wherein the learners repeatedly exchanged opinions and perspectives with fellow students to solve problems [30]. In this study, active self-directed learning helped participants in the experimental group to improve their reflective thinking, which reduces the gap between knowledge and practice. In the flipped learning context, existing evidence also suggests that preview lessons should be applied to achieve successful debriefing [17]. Thus, the combination of flipped learning and debriefing had desirable outcomes on the training applied in this study.

In sum, this study enabled student-directed debriefing through the use of video materials. A systematic review of the debriefing effects on simulation training in public-related education reported that debriefing with video materials had continuous educational effects on debriefing after the simulation, debriefing during the simulation, instructor-led debriefing, and instructor-led debriefing with video materials [31]. Future studies should therefore examine the effects of instructor-led debriefing with video materials.

### Limitations

In this study, all participants were sophomores at only one nursing college, which limited generalizability. However, it is worth noting that we implemented a new learning approach by combining debriefing and flipped learning in an in-school nursing practicum. Our positive results suggest that the same provisions should be adopted in other nursing courses to strengthen reflective thinking and practical skills among nursing students.

## 5. Conclusions

This study verified the effects of debriefing on nursing practice education via flipped learning among nursing students. The results showed that debriefing was effective for reflective thinking, but did not significantly impact academic motivation. Based on this, we propose the need to reinforce instructor roles and conduct replication studies with various targets to enhance the effects of debriefing.

## Figures and Tables

**Table 1:** Class time for the experimental and control groups.

Control Group	Experimental Group	Time
Flipped Learning	Adding Debriefing	
Quiz		10 min
Lecture and Demonstration		30 min
Group Practice	Group Practice and Debriefing	50 min
Summary		10 min

**Table 2:** Homogeneity between the experimental and control groups.

Characteristics	Categories	N (%) or M ± SD		χ^2^ or t(*p*)
		Experimental Group (n = 33)	Control Group (n = 30)	
Gender	Male	3 (9.1)	5 (16.7)	0.814 (0.367)
	Female	30 (90.9)	25 (83.3)	
Age (years)	Mean ± SD	20.79 ± 2.62	20.77 ± 2.47	0.33 (0.974)
Satisfaction with major	Strongly agree	19 (59.4)	14 (46.7)	1.405 (0.495)
	Agree	11 (34.4)	12 (40.0)	
	Neutral	2 (6.3)	4 (13.3)	
	Disagree	-	-	
	Strongly disagree	-	-	
Last semester grade	≥4.0	14 (46.7)	9 (31.0)	1.541 (0.463)
	3.0–3.9	15 (50.0)	19 (65.5)	
	2.0–2.9	1 (3.3)	1 (3.4)	
Flipped learning experience	Yes	3 (9.1)	8 (26.7)	4.100 (0.129)
	No	29 (87.9)	22 (73.3)	

**Table 3:** Pretest differences between the experimental and control groups.

Variables	Experimental Group (n = 33)	Control Group (n = 30)	t(*p*)
Motivation	3.73 ± 0.26	3.62 ± 0.91	1.917 (0.060)
Reflective Thinking	51.48 ± 5.48	49.77 ± 5.42	1.250 (0.216)

**Table 4:** Posttest differences between the experimental and control groups.

Variables	Experimental Group (n = 33)	Control Group (n = 30)	t(*p*)
Academic Motivation	3.71 ± 0.29	3.58 ± 0.26	1.864 (0.067)
Reflective Thinking	53.67 ± 5.71	50.80 ± 4.69	2.165 (0.034)

## Data Availability

Not applicable.
